# Removal of Methyl Violet from Aqueous Solution by Adsorption onto Halloysite Nanoclay: Experiment and Theory

**DOI:** 10.3390/toxics10080445

**Published:** 2022-08-03

**Authors:** Makfire Sadiku, Teuta Selimi, Avni Berisha, Arsim Maloku, Valbonë Mehmeti, Veprim Thaçi, Naim Hasani

**Affiliations:** 1 Department of Chemistry, Faculty of Natural and Mathematics Science, University of Prishtina, 10000 Prishtina, Kosovo; makfire.sadiku@uni-pr.edu (M.S.); teutaselimi3@gmail.com (T.S.); avni.berisha@uni-pr.edu (A.B.); arsim.maloku@uni-pr.edu (A.M.); veprim.thaci@uni-pr.edu (V.T.); 2 Faculty of Agriculture and Veterinary Medicine, University of Prishtina, 10000 Prishtina, Kosovo; valbonamehmeti1@gmail.com; 3 Department of Hydrotechnics, Faculty Civil Engineering, University of Prishtina, 10000 Prishtina, Kosovo

**Keywords:** halloysite, methyl violet, molecular modelling, monte carlo, adsorption

## Abstract

Methyl Violet (MV) was removed from aqueous solutions by adsorption onto halloysite nanoclay (HNC) employing equilibrium, kinetics, thermodynamic data, molecular modellingR (MD), and Monte Carlo (MC) simulations. The chosen experimental variables were pH, temperature, starting MV concentration, contact time, and adsorbent dosage. The adsorption rate was determined to increase with increasing contact time, initial dye concentration, pH, and temperature. The Langmuir, Freundlich, Temkin, and Dubinin–Radushkevich (D-R) isotherms were utilized to determine the adsorption capacity of HNC. The Langmuir equation matched equilibrium data better than the other models, whereas the pseudo-second-order model better described kinetic data, and thermodynamic analyses revealed that the adsorption process was spontaneous, endothermic, and physisorption-based. This study focused on two distinct molecular mechanics-based theoretical approaches (MC and MD). These techniques enabled a molecular comprehension of the interaction between the MV molecule and the halloysite surface. Theoretical results were consistent with experimental findings. The outcomes revealed that HNC is an excellent dye adsorbent for industrial effluents.

## 1. Introduction

Water is a crucial component for life, human evolution, and biodiversity conservation. Unfortunately, human activities have caused a serious degeneration in the quality of water resources. Even though a number of regulations are designed to ensure the safe disposal of industrial effluents, over 80 percent of the world’s wastewater is still disposed of without sufficient treatment [1,2,3]. The release of dye-containing effluents into the natural water bodies can cause harmful effects on the living systems because of their toxic, allergenic, mutagenic, and carcinogenic nature. Dyes obstruct light penetration, impede photosynthetic activity, and as a result, hinder the growth of biota and also cause micro-toxicity to fish and other organisms because of their predisposition to chelate metal ions [4,5].

In several industries such as food, plastic, textile, leather, paper, cosmetics, etc., organic dye effluent is one of the main water pollution problems [6]. Methyl violet (MV) is vital because of its extensive use in textiles, paints, and print inks, and the dying of cotton, silk, paper, and leather, among other applications [7]. MV is also used in medicine as an active ingredient in Gram’s biological stain for bacteria categorization [8,9,10,11]. Sometimes it can be used as a second-rate disinfectant but has been found to be toxic to most animals. Inhaling MV may irritate the respiratory tract, while ingesting it typically irritates the digestive tract [12,13].

Numerous technologies have been devised and implemented to eliminate synthetic dyes from aqueous solutions and limit their environmental impact [14,15,16,17,18]. Among the methods mentioned above, adsorption is better than the other techniques because of its low cost, simplicity of design, pliability, ease of working, and insensitivity to toxic pollutants [19,20]. Due to this, adsorption is currently recognized as a viable operation for the removal of dyes from the environment; it is a simple and economically viable procedure [21]. Materials such as walnut husk [22], modified chitosan composite [23], biochars from crop residues [24], natural zeolite [25], cross-linked succinyl chitosan [26], modified bentonite [27], quaternized poly(4-vinylpyridine) copolymers [28], natural clinoptilolite [29], activated carbon [30], chromium-intercalated montmorillonite [31], metal oxides [32,33,34,35], have been used to remove dyes from aqueous solutions. As well, alumina [36,37,38], silica gel [39,40], and clay minerals [41,42,43,44] are effective and they can support oxidizing environments.

Clay minerals are low-cost, exist in abundance, and are ordinarily innoxious for environmental applications. Their large specific surface area, high porosity, surface charge, and surface functional groups enable the use of clay minerals as adsorbents, flocculants, and filtration media [45,46]. It was discovered that the adsorption capabilities of clays may be greater than those of activated carbon at the same temperature and pH conditions [47]. Adsorption and desorption of organic compounds on clays are primarily determined by the interfacial characteristics of these materials and the chemical properties of their molecules [48]. Clays have a high adsorption capacity for positively charged cations such as cationic dyes, heavy metals, etc., due to their net negative charge.

In this work, the removal of MV from aqueous solutions by adsorption utilizing HNC as an adsorbent was examined. Compared to the earlier published research on the adsorption of MV by HNC published by Liu et al. [49], this work explores the experimental part in more depth and employs theoretical calculations.

The Langmuir, Freundlich, Temkin and Dubinin Radushkeviq (D-R) equations were used to fit the equilibrium isotherms. A kinetic study was carried out with first-order, pseudo first-order, second-order, pseudo second-order, intraparticle and film diffusion models. The thermodynamic parameters derived from adsorption experiments in the present work are extremely useful for elucidating the nature of MV adsorption on halloysite. On the molecular level, the theoretical calculations are of utmost importance as they offer the possibility to evaluate the interaction of molecules on the surfaces. MC and MD provided insights into the theoretical description of the interaction between MV and HNC surface at a molecular level. 

## 2. Materials and Methods

### 2.1. Adsorbent and Adsorbate

In this study commercial halloysite nanoclay (Al_2_Si_2_O_5_ (OH)_4_ × 29H_2_O) supplied by Aldrich, was used as an adsorbent.

As adsorbate, Methyl Violet dye purchased from Fluka was utilized and was used without further purification. The stock solution (1 g L^−1^) was prepared by dissolving 1 g of the dye into distilled water. The experimental dye solution was made by diluting the stock solution with the necessary amount of distilled water.

### 2.2. Batch Experimental Procedure

Batch experiments were performed by stirring 25 mL of known concentration of MV solution with 0.2 g of HNC using magnetic stirrer. 

Several parameters such as effect of contact time (1–240 min), effect of initial concentration (100, 125, 150, 175, and 200 mg/L), effect of pH (4–10), and effect of temperature (11, 25, and 40 °C) were done for optimizing the experimental conditions. 

The pH was adjusted using 0.1 M HCl and 0.1 M NaOH. 

After adsorption, the adsorbent and the supernatants were separated by centrifugation at 5000 rpm for 10 min and samples were investigated for residual dye concentration using a UV–Visible Spectrophotometer (type T70+), at wavelength 579 nm, using a 1 cm quartz cell.

All experiments were repeated three times and mean values were presented with a maximum deviation of 5%.

The amount of MV adsorbed per unit adsorbent (mg/g) was calculated using Equation (1):

(1) $$q_{e}=\frac{\left(C_{i}-C_{e}\right)*V}{m}\;\;$$

where *C_i_* is the initial dye concentration (mg/L), *C_e_* is the equilibrium dye concentration (mg/L), *V* is the volume of MV solution used (L), and *m(g)* is the mass of HNC used.

The MV percent removal was calculated using Equation (2).

(2) $$Removal\;\left(\%\right)=\frac{\left(C_{i}-C_{e}\right)*100}{C_{i}}\;$$

To construct and evaluate an adsorption process, it is necessary to fit equilibrium adsorption data with various adsorption isotherm and kinetic models. Thus, several theoretical models (Table 1) are used for experimental data in order to identify a model that predicts kinetic and isotherm data adequately. The validity of the models was determined by calculating the regression coefficient (R^2^) and the root mean square errors (*RMSE*):

(3) $$RMSE=\sqrt{\frac{\Sigma {\left(q_{exp-}q_{cal}\right)}^{2}}{N}}$$

where *q_exp_* is the experimental value while *q_cal_* is the calculated value and *N* is the number of observations in the experiment. The smaller the *RMSE* values, the better the curve fitting [50,51]. Exel 2016 was used to create all of the graphs.

### 2.3. Molecular Modelling and Monte Carlo Calculations

#### 2.3.1. MV and HNC Molecular Modelling 

**Figure 1:**
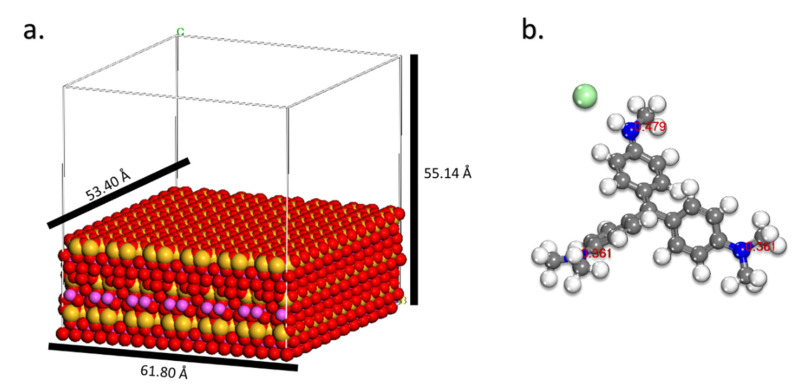
Molecular models used in the theoretical calculations: (**a**) HNC surface, (**b**) MV molecule (with MAC charges).

For the interaction amongst the HNC surface and the MV molecule (Figure 1) in the Monte Carlo (MC) and Molecular dynamic (MD) calculations, the simulation was done using the halloysite model (under Periodic Boundary Condition) with cell size of: 61.80 Å × 53.40 Å × 30.14 Å with the inclusion of a 25 Å vacuum layer at C axis containing inside 2500 water molecules and 1 MV molecule.

#### 2.3.2. Monte Carlo and Molecular Dynamic Simulation Details 

Six cycles of simulated annealing, each with 15,000 steps, were used to do the MC computations. The annealing temperature was automatically selected between 10^5^ and 10^2^ K for each cycle. As the temperature steadily decreased, potential adsorption structures were uncovered [52,53]. The MD is performed under an NVT ensemble at 25 °C with a 1 fs time step and 1 ns total simulation period. The temperature control is realized using the Berendsen thermostat MD [54]. MC and MD simulations use COMPASS II’s force field. Radial Distribution Function (RDF) is computed using 1 ns of MD trajectory [55,56].

## 3. Results and Discussion

### 3.1. Effect of pH 

**Figure 2:**
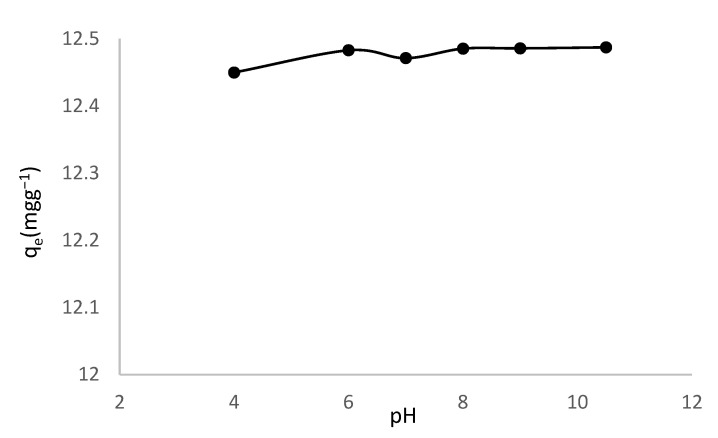
Effect of pH on adsorption of MV onto HNC.

Solution pH affects adsorption. It affects the adsorbent’s surface charge, the solution’s ionization, and the dissociation of functional groups on the active sites, as well as solution dye chemistry [57,58]. Figure 2 shows the effect of pH on MV removal effectiveness from 4.0 to 10.5. As illustrated in Figure 2, the pH rise from 4 to 10.5, has an insignificant effect on the adsorption capacity. This is in line with other studies such as the adsorption of methyl violet on halloysite nanotube [49] and the adsorption of MG on treated ginger waste [59].

### 3.2. Effect of Contact Time and Initial Concentration

**Figure 3:**
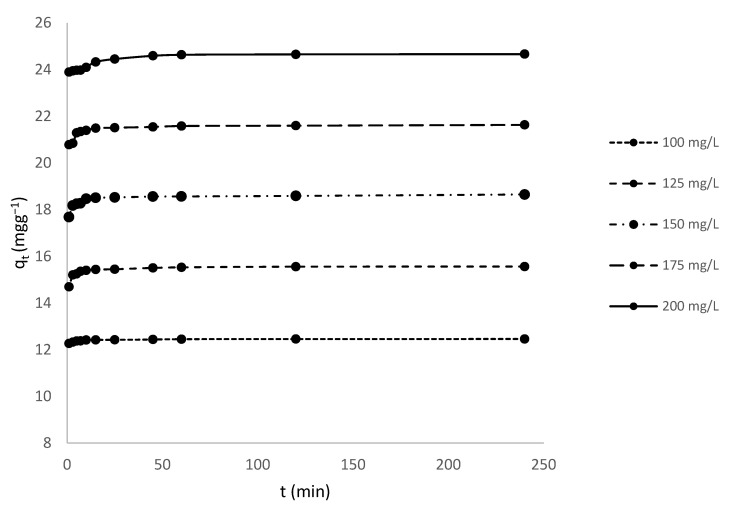
Time and dye concentration affect MV adsorption on HNC.

In Figure 3, the effects of the initial concentrations of MV (100, 125, 150, 175, and 200 mg/L) and contact time (1–240 min) on the adsorption capacity of HNC at 298.15 K are shown. 

MV adsorption was faster in the first 10 min, but slowed when the halloysite became saturated, reaching equilibrium after 240 min. High beginning adsorption rates may be owing to a large number of binding sites, whereas lower rates at the end are due to saturation and equilibrium. Similar conclusions have been proposed [60] for the adsorption of MG by neem sawdust.

Also, adsorption increases with increasing initial dye concentration. According to the results obtained, adsorption capacity increased from 12.46 mg/g to 24.66 mg/g as the initial concentration of MV dye increased from 100 mg/L to 200 mg/L. As initial concentration increases, mass transfer driving force overcomes resistances to dye molecule mass transfer from solution to the solid phase. Also, increasing concentration promotes dye adsorption by increasing dye-halloysite interaction [61,62].

### 3.3. Effect of Adsorbent Dosage

**Figure 4:**
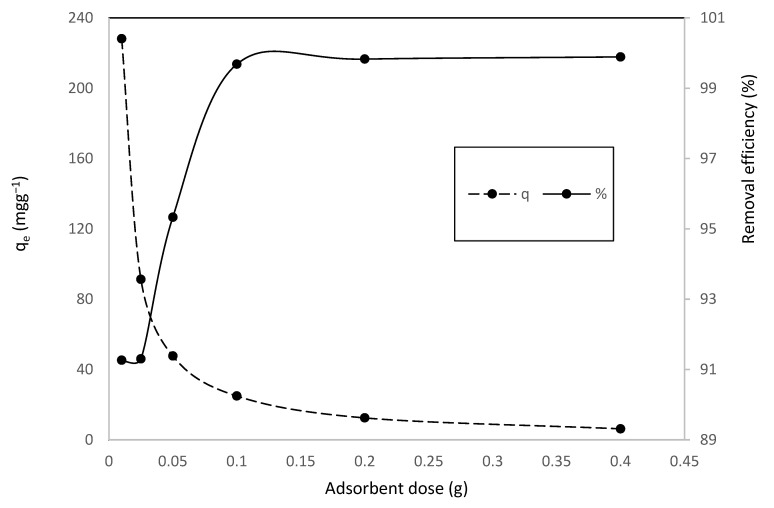
Effect of adsorbent dosage on adsorption of of MV onto HNC.

It was noticed (Figure 4), that the percent of MV adsorption grew significantly from 91–99.9%, with the increase of adsorbent dosage from 0.025 to 0.2 g. Thereafter, by increasing the adsorbent dosage up to 0.4 g, an insignificant increase in the removal efficiency was observed [63].

It was also found that the enhancement of adsorbent dosage, resulted in a decrease in the amount of adsorbed dye per unit mass of adsorbent, from 91.3 to 6.3 mg/g.

Due to a divide in the concentration gradient between dye concentration in the solution and dye concentration on the HNC surface, *q_e_* (mg/g) decreases with increasing adsorbent mass [64].

A similar way of behaving was reported for mercury (II) removal on EB [65] and MB adsorption on guava leaf [66], on gulmohar plant leaf [67], and on cashew nutshell activated carbon [68].

### 3.4. Effect of Temperature

**Figure 5:**
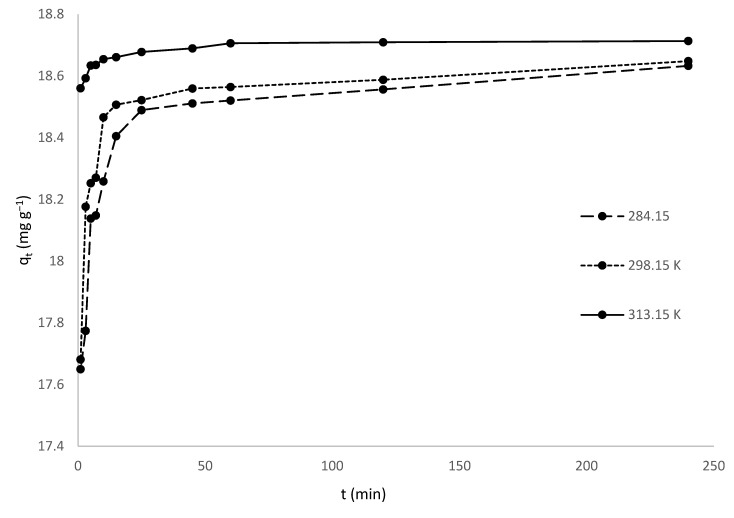
Effect of temperature on adsorption of MV onto HNC.

The effect of temperature on MV dye adsorption onto HNC is introduced in Figure 5.

The equilibrium adsorption capacity of MV onto HNC was found to increase slightly with increasing temperature, from 18.63 mg/g at 284.15 K to 18.71 mg/g at 313.15 K. With increasing temperature, the viscosity of the solution decreases, and consequently the rate of diffusion of dye molecules increases. This leads to an increase in mobility of molecules from the bulk solution to the surface of the adsorbent, and as a result, an increase in the amount of MV adsorbed [69,70].

A similar temperature effect was reported for adsorption of MV adsorption by clinoptilolite [71] and adsorption of the dyestuff astrazon red violet 3 rn (basic violet 16) on montmorillonite clay [72]. 

### 3.5. Adsorption Isotherms

Four isotherm equations specifically Langmuir, Freundlich, Temkin and Dubinin–Radushkevich (D-R) (Table 1) were employed to interpret the experimental data for MV adsorption on HNC at different temperatures.

The results are shown in Table 2.

Based on the R^2^, RMSE values, *q_e,calc_* values, and modelled isotherms, the Langmuir isotherm model fitted best amongst all the isotherm models investigated. Langmuir isotherm model assumes similar, energetically equivalent adsorption sites and monolayer adsorption [73]. 

The Langmuir isotherm constants, *K_L_* and *q_m_*, were calculated from the slope and intercept respectively, of linear plot of *C_e_/q_e_* against *C_e_* (Figure 5 and Table 2).

The separation factor (*R_L_*), an essential parameter of the Langmuir isotherm, is generally used to show if the adsorption process is unfavorable (*R_L_* > 1), linear (*R_L_* = 1), favorable (0 < *R_L_* > 1), or irreversible (*R_L_* = 0). It can be calculated by Equation (4).

(4) $$R_{L}=\frac{1}{1+K_{L}C_{i}}\;\;$$

The values of *R_L_* in Table 2 indicate that the adsorption of MV on HNC is favorable.

The Freundlich isotherm which assumes multilayer sorption on the heterogeneous adsorbent surface was also utilized to evaluate experimental data. *K_F_* and 1/*n* were calculated from the intercept and slope of the linear plot of log *q_e_* versus log *C_e_*. The values of n greater than unity in Freundlich adsorption isotherm, for all temperatures, indicated that the HNC is suitable for the adsorption of MV. 

The Temkin isotherm model includes a factor that takes into account the interactions between the adsorbent and adsorbate. The heat of molecular adsorption in the layer would reduce due to these interactions and the adsorption is identified by a dissipation of binding energies. The determined Temkin parameters (*b_T_* and *K_T_*) exhibited that the interactions between the adsorbent surface and the MV dyes are weak and may be a physical adsorption process. The increased value of *b_T_* with temperature also supports increased adsorption efficiency with increasing temperature [74,75].

Dubinin-Radushkevich (D-R) isotherm model determines whether adsorption is physical or chemical. The values of constants q_m_ and β are given in Table 2. The parameter β could be used to calculate the mean free energy (E = 1/√2β), which could distinguish the type of the adsorption process. If the value of E is less than 8 kJ mol^−1^, the adsorption process is physical, and when E is between 8 kJ mol^−1^ and 16 kJ mol^−1^, the process is chemical adsorption [76].

The value of apparent energy E (less than 8.0 kJ mol^−1^) indicated that the adsorption of MV onto the HNC is a physical process and is consonant with the ∆G parameter results [77].

**Figure 6:**
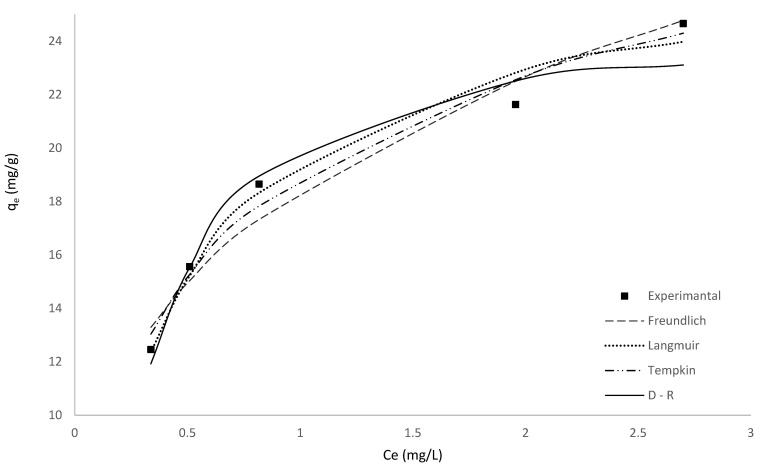
Comparison of the experimental and modelled isotherms for the adsorption of MV by HNC.

The nonlinear relationship of the isotherm models used, is shown in Figure 6.

### 3.6. Adsorption Kinetics

In order to investigate the adsorption process and potential rate controlling step of MV adsorption onto HNC first order, pseudo-first-order, second order, pseudo-second-order, and intra-particle diffusion kinetic models were studied (Table 1). 

**Figure 7:**
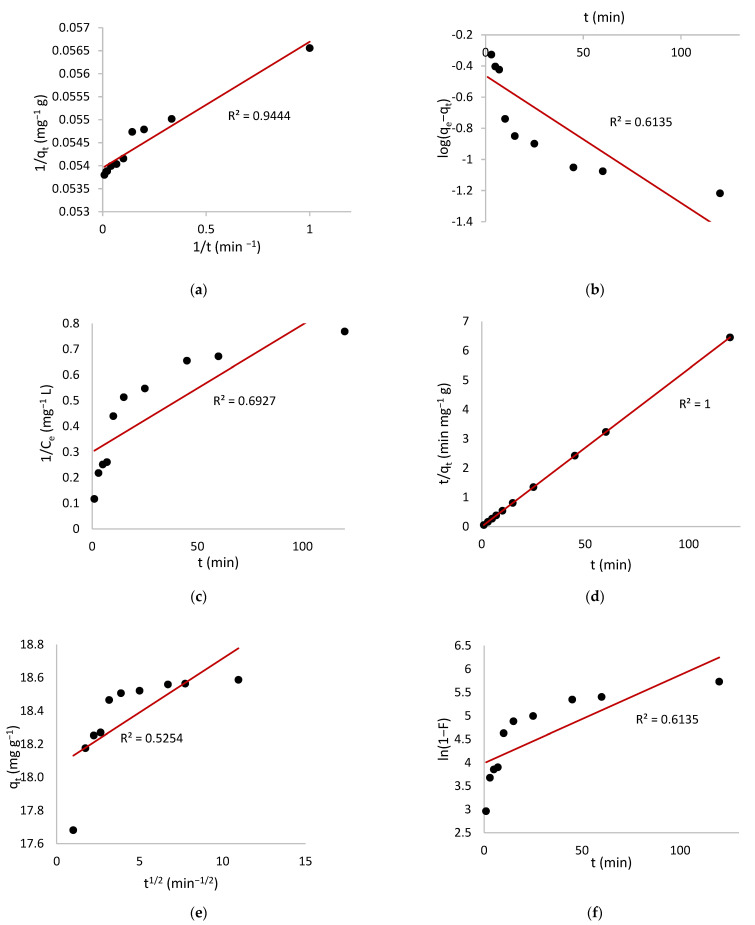
Kinetic model plots for MV adsorption onto HNC (**a**) first order, (**b**) pseudo-first order, (**c**) (second order, (**d**) pseudo-second order, (**e**) intraparticle diffusion and (**f**) liquid film diffusion.

Figure 7 shows the linear relationship of kinetic models for the MV adsorption process and the value of kinetics parameters determined using these models are given in Table 3.

On the basis of the high values of the regression coefficient, (R = 1) and very close values of *q_e,calc_* with *q_e,exp_*, the pseudo-second-order rate model fitted the experimental data better than any other model studied, providing evidence that the adsorption of MV on HNC followed the pseudo-second-order kinetic model. Considerable reduction of dye amount during batch adsorption experiments and longer time needed for adsorbing species to diffuse to remote locations deep within a network of fine pores are the two contributing explanations for these results [79,80].

Similar findings are also reported for adsorption of methylene blue by coconut husks/polylactide blended films [81], adsorption of CV dye on zeolites from coal fly and bottom ashes [78], and adsorption of CV dye on coffee husks [82].

Adsorbate molecules travel from the aqueous phase to the adsorbent surface, then diffuse into pores. External mass transfer (boundary layer diffusion) or intraparticle diffusion determines the adsorption rate. The experimental adsorption kinetics data were fitted using the intraparticle diffusion model and the liquid film diffusion model to determine the rate-determining step.

If the intraparticle diffusion is the sole rate-limiting step, the plot of q_t_ vs. t^0.5^ gives a straight line with zero intercept. As the intraparticle diffusion plot did not pass through the origin some other mechanism might be included. For that reason, the kinetic data were examined by the liquid film diffusion model. Linear graphs of ln (1 − F) vs. t with zero intercept suggest that adsorption kinetics are driven by diffusion through the liquid film surrounding solid adsorbents. The low values of *R*^2^ (Table 4) and the non-zero intercept plots of the liquid film diffusion model indicate that the liquid film diffusion is also not the sole rate-determining step. 

So the adsorption mechanism is a combination of the two processes mentioned above [51,77].

### 3.7. Adsorption Thermodynamics

The free energy change (Δ*G*^0^), enthalpy change (Δ*H*^0^), and entropy change (Δ*S*^0^) were evaluated using the following equations:

(5) $$\Delta G^{0}=-RT\;lnK_{c}$$

(6) $$lnK_{c}=\frac{\Delta S^{0}}{R}-\frac{\Delta H^{0}}{RT}$$

(7) $$K_{c}=K_{L}\times 10^{6}$$

where *Kc* is the equilibrium constant (dimensionless), [83,84] *R* is the gas constant (J/Kmol), and *T* is the temperature (K).

**Figure 8:**
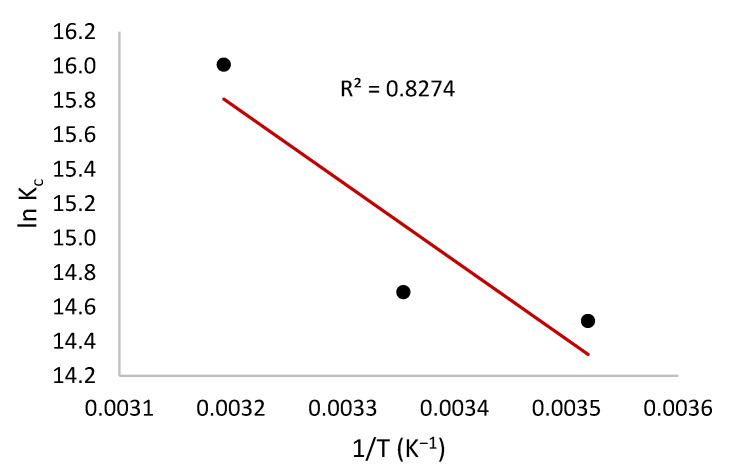
Plot of ln *Kc* versus 1/*T* of the adsorption process.

The Δ*H*^0^ and Δ*S*^0^ were determined from the slope and intercept of the plot of *lnK_c_* versus 1/T (Figure 8), and the results are shown in Table 5.

The positive value of Δ*H*^0^ (+37.84 kJ/mol) indicated that the adsorption process was endothermic and since this value is less than 80 kJ/mol (80 kJ/mol is the upper limit of the change of enthalpy for physisorption), it shows that the adsorption follows a physisorption.

The positive value of Δ*S*^0^ indicated increased disorder and randomness at the solid-liquid interface; however, the negative value of Δ*G*^0^ suggested spontaneous and thermodynamically favorable adsorption of MV onto HNC. 

Meantime, the value of Δ*G*^0^ became more negative with increasing temperature, which exhibits that higher temperature is contributory to adsorption [19].

The activation energy *Ea* was obtained using the Arrhenius equation:

(8) $$lnk_{2}=lnA-\frac{E_{a}}{RT}$$

where *k*_2_ is the pseudo-second-order rate constant, *A* is the Arrhenius constant, *E_a_* refers to the energy of activation (J mol^−1^), *R* is the ideal gas constant (8.314 J mol^−1^ K^−1^), and *T* is the temperature (K).

**Figure 9:**
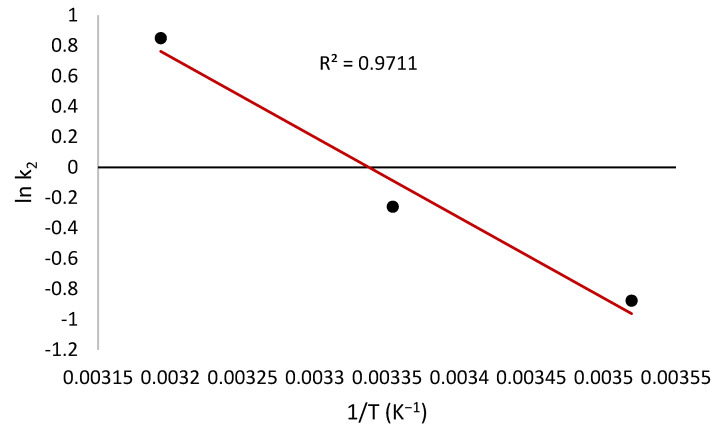
Plot of ln *k*_2_ versus 1/*T* of the adsorption process.

The slope of the plot of *ln k*_2_ versus 1/*T* (Figure 9) is used to evaluate *E_a_*, which was found to be 44 kJ/mol.

This value is consistent with the values of the activation energy (43.0 kJ/mol) for the adsorption of reactive red 189 on cross-linked chitosan beads [85], and (5.6–49.1 kJ/mol) for the adsorption of polychlorinated biphenyls on fly ash [86].

### 3.8. Monte Carlo and Molecular Dynamic Simulations

**Figure 10:**
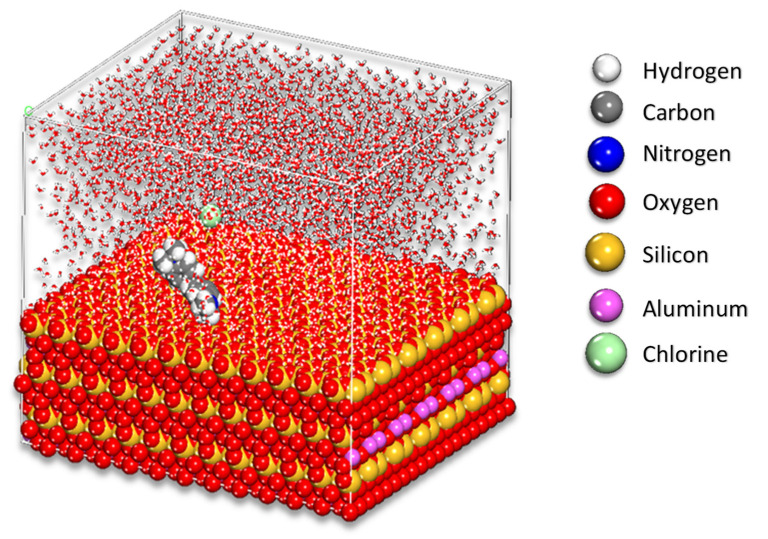
Final MC poses of the lowest adsorption configurations for the MV in the simulated corrosion media on the HNC surface PBC model.

Figure 10 depicts the lowest energy configurations for the MV on the HNC surface under the simulated adsorption circumstances that were employed (as chosen above). As evidenced by Mulliken charges (Figure 1), the adsorption geometries of the MV demonstrate that nitrogen atoms are mostly responsible for this behavior.

The following equation may be used to calculate the adsorption energies of the adsorbate molecule on the halloysite surface.

(9) $$E_{ads}=E_{total}-\left[E_{surface+water}+E_{MV+surface}\right]+E_{water}$$

where: *E_total_* is the total energy of the system as a result of adsorbent-adsorbate; *E_surface+water_* and *E_MV+water_* is system energy in the absence and presence of MV.

**Figure 11:**
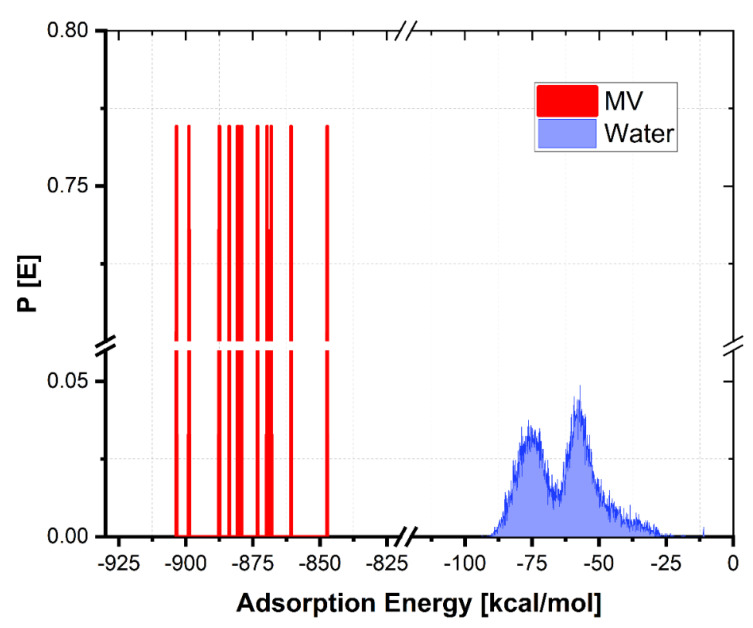
Distribution of adsorption energies for MV onto the halloysite surface.

Figure 11 depicts the distribution of the adsorption energies for the large number of adsorptive configurations developed and computed by the Monte Carlo approach for the MV adsorbate, which was based on the results of the Monte Carlo method.

The Adsorption Energy (*E_ads_*) values for the adsorption of MV onto the HNC surface are presented in Table 6.

**Figure 12:**
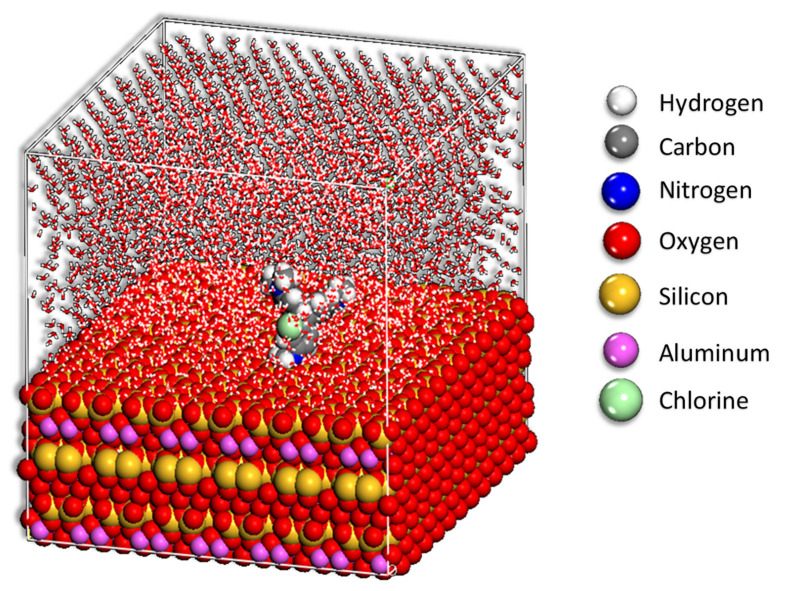
Final MD pose of the lowest adsorption configurations for the MV in the simulated corrosion media on the HNC surface PBC model.

As indicated by the high *E_ads_*, the MV has a strong contact with the halloysite surface, resulting in good adsorptive properties. Adsorption of MV onto the halloysite surface was determined by both the MC and MD calculations, respectively. In the case of the adsorption process, Monte Carlo simulations (see Figure 10 and Figure 12) have indicated that the ensuing negative values of adsorption are indicative of the spontaneity with which the process occurs (Figure 11). It is important to employ MD simulations because they give a very simple means of tracking and recording the kinetics of inhibitor adsorption on the metal surface under study. Figure 12 shows the final arrangements of MV on the halloysite surface as depicted in the previous figure.

The presence of an RDF peak between 1 and 3.5 Å from the surface plane of the material and the adsorbate atom confirms adsorption, but for physisorption, RDF peaks at greater distances confirm physisorption, and vice versa.

**Figure 13:**
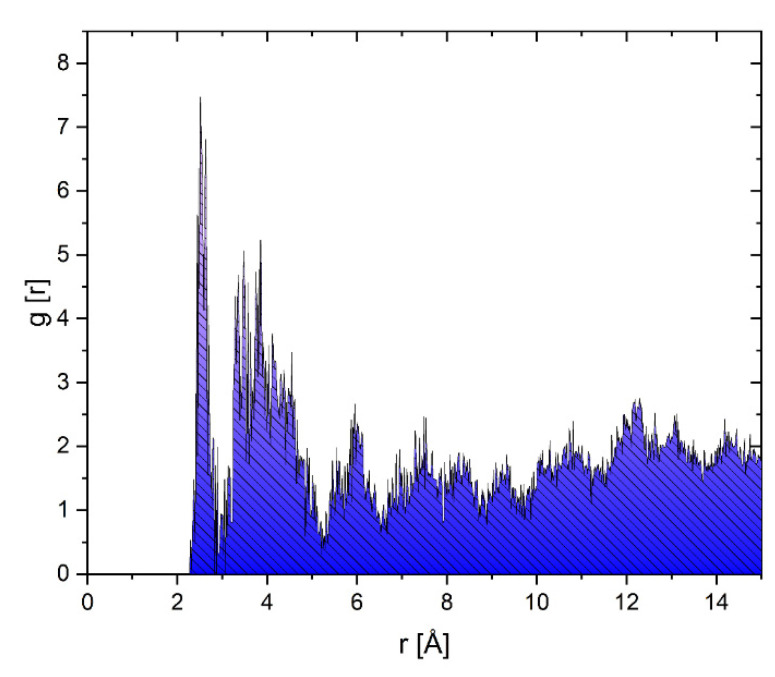
RDF of nitrogen MV the halloysite surface obtained via MD.

The RDF of the nitrogen atoms in the MV (Figure 13 is close to the surface plane, suggesting that these components have an increased interaction with the adsorbent surface. The findings of the MD and RDF analyses confirm that these inhibitors have a high proclivity to adsorb onto the surface, owing to their peculiar attraction to share and accept electrons with the surface in question. [55]. 

### 3.9. FTIR Spectroscopy

**Figure 14:**
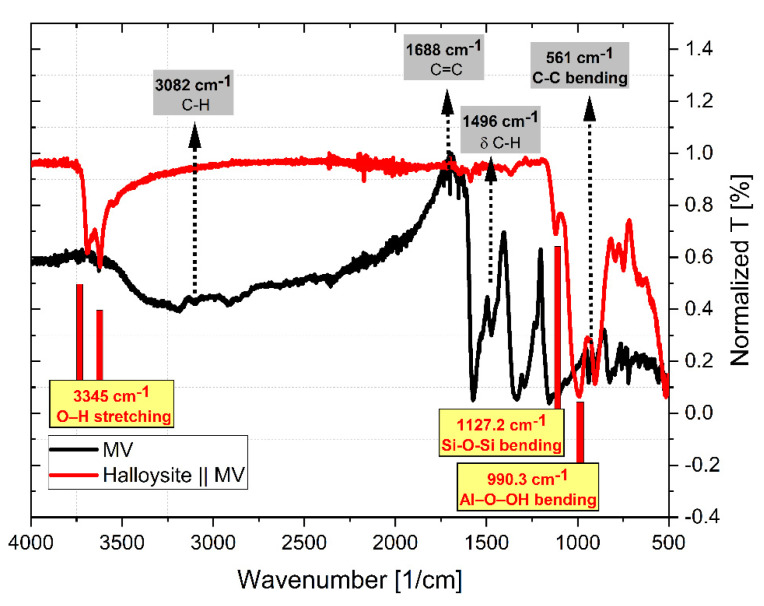
The FTIR spectrum of MV and MV-HNC.

FTIR-8400S was used for Total Attenuated Reflection (ATR) measurements with the following parameters: resolution 2 cm*^−^*^1^, 100 scans, 500–4000 cm*^−^*^1^. Figure 14 exhibits the peaks that appeared at certain wave numbers of MV FTIR spectra before and after adsorption onto HNC.

The following peaks: 3082 and 1496 cm^−1^ show -CH- aromatic and 2927 cm^−1^ –CH_3_ methyl vibrating vibrations, respectively 1688 cm^−1^, aromatic ring vibrations –C=C– vibrations from the skeleton of aromatic ring structures of MV, while we have new displays of vibrations of the group -OH at 3345 cm^−1^ stretching, 1127.2 cm^−1^ bending by the group connections -Si-O-Si- bending and 990.3 cm^−1^ from the -Al-O-OH- bond arising from the Halloysite and MV groups with distinctive features (as shown in Figure 14).

The interaction of MV molecules with Halloysite functional groups was confirmed by the emergence and disappearance or depletion of various peaks.

## 4. Conclusions

This study established the viability of halloysite nanoclay as a dye-removal adsorbent in wastewater. The findings of the batch trials revealed that adsorption was affected by adsorbent dose, contact time, initial dye concentration, and temperature.

Adsorption of MV is very little affected by changing the temperature and pH. The optimal adsorption capacity (27.7 mg/g) was reached at ambient temperature (298 K) and pH (4.26). 

Among the investigated isotherm models, the Langmuir isotherm model demonstrated the best fit.

The experimental data were best suited by the pseudo-second-order rate model, indicating that the adsorption of MV on HNC followed the pseudo-second-order kinetic model. The kinetic analysis indicated that the adsorption mechanism is a combination of intra-particle diffusion and film diffusion.

Thermodynamic investigations demonstrated that the removal of MV by HNC is possible, exothermic, and spontaneous.

This study focuses on two distinct molecular mechanics-based theoretical approaches (MC and MD). These techniques enabled a molecular comprehension of the interaction between the MV molecule and the halloysite surface. Simulations using Monte Carlo and molecular dynamics revealed that this molecule spontaneously adsorbs onto surfaces. The negative value of the adsorption energies supports a strong interaction between the MV and HNC surface, which is consistent with experimental findings.

## Figures and Tables

**Table 1:** Isotherm, kinetic, and other equations used in this study.

Model	Equation	Parameters
Isotherm models		
Langmuir	$\frac{C_{e}}{q_{e}}=\frac{1}{q_{m}K_{L}}+\frac{C_{e}}{q_{m}}$	*q_e_* (mg g^−1^)—equilibrium adsorption capacity
		*q_m_* (mg g^−1^)—maximum adsorption capacity
		*K_L_* (L mg^−1^)—Langmuir constant *C_e_* (mg L^−1^)—equilibrium dye concentration
Freundlich	$lnq_{e}=lnK_{F}+\frac{1}{n}lnC_{e}$	*K_F_*—(mg g^−1^) (L g^−1^)^1/n^—Freundlich constant *n*—heterogeneity factor
Temkin	$q_{e}=\frac{RT}{b_{T}}lnK_{T}+\frac{RT}{b_{T}}lnC_{e}$	*K_T_* (L g^−1^)—Temkin constant related to adsorption capacity. *b_T_* (J mol^−1^)—Temkin constant related to the heat of sorption
Dubinin–Radushkevich (D-R)	$\begin{array}{c} lnq_{e}=lnq_{m}-\beta \varepsilon^{2}\;\;\\ \varepsilon =RTln\left(1+\frac{1}{C_{e}}\right) \end{array}$	*β* (mol^2^/J^2^): D-R constant *ε* (J mol^−1^): Polanyi potential *R* (8.314 J mol^−1^ K^−1^)—universal gas constant *T* (K)—temperature
Kinetic models		
First-order	$\frac{1}{q_{t}}=\frac{1}{q_{e}}+\frac{k_{1}}{q_{e}t}$	*q_t_* (mg g^−1^): amount of adsorbate adsorbed at time *t* *k*_1_ (min^−1^): first-order rate constant
Pseudo-first-order	$\ln\left(q_{e}-q_{t}\right)=ln\;q_{e}-k_{1}t$	*k*_1_ (min^−1^): pseudo-first-order rate constant
Second-order	$\frac{1}{C_{e}}-\frac{1}{C_{0}}=k_{2}t$	*k*_2_ (L mg^−1^ min^−1^): second-order rate constant
Pseudo-second order	$\frac{t}{q_{t}}=\frac{1}{k_{2}q_{e}^{2}}+\frac{t}{q_{e}}$	*k*_2_ (g mg^−1^ min^−1^): pseudo-second-order rate constant
Intraparticle-diffusion	$q_{t}=K_{i}t^{1/2}+C$	*k_i_* (mg g^−1^ min^−0.5^): intraparticle diffusion rate constant
Liquid film diffusion	$\begin{array}{c} \ln\left(1-F\right)=k_{fd}t+C\\ F=\frac{q_{t}}{q_{e}} \end{array}$	*F*—fractional attainment of equilibrium, equal to *q_t_*/*q_e_*

**Table 2:** Isotherm parameters for the adsorption of MV onto HNC.

Model	Parameters				
	Equation		284.15 K	298.15 K	313.15 K
Langmuir	$q_{e}=\frac{q_{m}bC_{e}}{1+bC_{e}}$	*q_m_* (mg g^−1^) *K_L_* (L mg^−1^) *R_L_* *R*^2^ *RMSE*	27.8550 2.0200 0.0025 0.9990 0.4700	27.7000 2.3900 0.0021 0.9940 0.6500	25.3800 8.9500 0.0006 0.9998 0.7000
Freundlich	$q_{e}=K_{F}C_{e}{}^{1/n}$	*K_F_* (mg g^−1^) *n* *R*^2^ *RMSE*	17.5700 3.3200 0.9600 0.9200	18.4000 3.3300 0.9575 0.8000	21.0800 5.3600 0.8086 1.9900
Temkin	$q_{e}=\frac{RT}{b_{T}}\ln(K_{T}C_{e})$	*K_T_* (L mg^−1^) *RT*/*b* (kJ/mol) *b_T_* (J mol^−1^) *R*^2^ *RMSE*	27.9300 5.4310 435.0000 0.9834 0.5500	32.7200 5.4220 457.1800 0.9770 0.6400	529.1100 3.4400 755.7800 0.8642 9.7100
D-R	$q_{e}=q_{m}\exp\left(-\beta \varepsilon^{2}\right)$	*q_m_* (mg g^−1^) β E (kJ/mol) *R*^2^ *RMSE*	23.0100 7 ×10^−8^ 2.6720 0.9402 1.1600	23.9700 6 × 10^−8^ 2.890 0.9761 0.8400	24.8300 2 × 10^−8^ 5.000 0.9811 6.6000

**Table 3:** Comparison of HNC adsorption capacities with various adsorbents.

Adsorbent	q_max_/mg g^−1^	Ref
Natural and Zwitterionic Surfactant-modified Clay	54.61	[1]
Halloysite Nanotubes	187.18	[46]
DTMA-bentonite	740.5	[48]
Lignit coal	40.82	[50]
Halloysite Nanotubes	97.96	[75]
Halloysite Nanotubes	84.32	[78]
Na-bentonite	67.1	[48]
Magnetic composite	20.04	[13]
HNC	27.7	This study

**Table 4:** Kinetic parameters for the sorption of MV onto HNC.

Model	*q_e,exp_* (mg g^−1^)	Parameters	
First-order	18.648	*k*_1_ (min^−1^) *q_e,calc_* (mg g^−1^) *R*^2^	0.0500 18.5200 0.9444
Pseudo first-order		*k*_1_ (min^−1^) *q_e,calc_* (mg g^−1^) *R*^2^	0.0188 0.3450 0.6135
Second-order		*k*_2_ (g mg^−1^ min^−1^) *R*^2^	0.0050 0.6927
Pseudo second-order		*k*_2_ (gmg^−1^ min^−1^) *q_e,calc_* (mg g^−1^) *R*^2^	0.6300 18.5900 1
Intrapaticle diffusion		*k_i_* (mg g^−1^ min^−1/2^) *C* *R*^2^	0.0650 18.0700 0.5254
Liquid film diffusion		*K_fd_* C *R*^2^	0.0188 3.9895 0.6135

**Table 5:** The thermodynamic parameters of adsorption of MV onto HNC.

Temperature (K)	Δ*G*^0^ (kJ/mol)	Δ*H*^0^ (kJ/mol)	Δ*S*^0^ (J/mol K)
313.15	−41.68	37.84	252.30
298.15	−36.40		
284.15	−34.30		

**Table 6:** The distribution *E_ads_* values for MV onto the Halloysite surface.

Molecule	Adsorption Media		
	Min.	Max.	Mean Value
**MV**	−847.15	−903.55	−875.35

## Data Availability

Not applicable.
